# White-coat hypertension detected during opportunistic blood pressure screening in a dental healthcare setting

**DOI:** 10.1080/02813432.2021.1958496

**Published:** 2021-08-04

**Authors:** Helen Andersson, Lennart Hedström, Håkan Bergh

**Affiliations:** a Hallands Hospital, Varberg, Sweden; b Department of the Institute of Medicine, University of Gothenburg, Gothenburg, Sweden; c Public Dental Services, Västra Vall, Varberg, Sweden; d Department of Research and Development Unit, Hallands Hospital, Varberg, Sweden

**Keywords:** White-coat hypertension, blood pressure, home blood pressure, screening, dental health care

## Abstract

**Objective:**

To study white-coat hypertension (WCHT, blood pressure ≥140/90 mmHg in a clinic and normal blood pressure <135/85 mmHg at home), with blood pressure screening of a healthy population during their dental healthcare visit and the associated risk factors.

**Design:**

A multicentre observational study.

**Setting:**

A healthy general population at four dental clinics in a region in southern Sweden.

**Subjects:**

2025 individuals aged 40–75 years were screened for high blood pressure at their annual regular check-up dental visit.

**Main outcome measures:**

Frequencies of normal and elevated blood pressure (BP) in dental clinics, with home BP as a reference. According to BP results, the population was divided into three groups: normotension (NT), WCHT and suspected hypertension (HT). Background and life style factors were measured: sex, age, family history of hypertension, body mass index (BMI kg/m^2^), education level, tobacco use, and physical activity level.

**Results:**

The overall prevalence of WCHT in the study was 17.7%, and the prevalence was 57.2% among those with clinically high blood pressure. Compared with NT, WCHT was associated with male sex (OR 1.56, CI 1.18–2.06), older age group (OR 2.33, CI 1.66–3.26), family history of hypertension (OR 1.61, CI 1.24–2.10), high BMI kg/m^2^ (OR 2.36, CI 1.80–3.10), daily snuff use (OR 1.74, CI 1.19–2.53). In comparison with WCHT, HT was associated with male sex (OR 2.16, CI 1.44–3.25), older age group (OR 2.85, CI 1.75–4.65), daily smoking (OR 2.10, CI 1.14–3.85), less daily snuff use (OR 0.59, CI 0.34–0.99).

**Conclusions:**

The prevalence of WCHT in a healthy population was 17.7%. Regarding cardiovascular risk factors, WCHT seems to be in the middle of NT and HT. Individuals with WCHT can be identified and given lifestyle advice in connection with a dental check-up, but follow-up and assessment of their cardiovascular risk should take place in primary care.

**Key points**
- Screening in dental practice can detect white-coat hypertension (WCHT) (17.7%) and suspected hypertension (HT) (12.4%).
- Individuals with WCHT have more cardiovascular risk factors than normotensive individuals.
- Individuals with WCHT could be given lifestyle advice in dental clinics according to current guidelines.

## Introduction

Hypertension is a very common risk factor worldwide; it is the most important risk factor for total disease burden in the world, and it is strongly associated with cardiovascular diseases [1]. Hypertension sequelae cause much suffering for affected individuals and a large cost to society. In Sweden, it is estimated that 27% of the adult population has high blood pressure [2]. As high blood pressure is often asymptomatic, up to 50% of those affected are unaware they have hypertension [1–3]. Hypertension is best detected by structured population screening programmes or opportunistic screening measurements of blood pressure (BP), and according to guidelines, all adults should have their BP recorded at regular intervals with the frequency dependent on their BP level [4].

Healthy lifestyle choices can prevent or delay the onset of hypertension and can reduce metabolic risk factors [4].

### Screening

While early detection by means of systematic screening programmes is not currently performed in Sweden, systematic BP screening occurs among individuals at risk (diabetes, cardiovascular disease, and obesity) within the primary health care (PHC) system. Those who have no chronic illnesses usually seek PHC for acute conditions (infection, injury, and life crisis), and often it is inappropriate to measure BP, which is due to these acute situations. Since a majority of the healthy population (80% in Sweden) regularly seeks dental care services [5], dental care service providers can be possible providers of opportunistic screening for common risk factors such as hypertension [6–8].

### White-coat hypertension

White-coat hypertension (WCHT) refers to the untreated condition in which BP is elevated in the office (≥140/90 mmHg) but is normal when measured by ambulatory (<130/80 mmHg) or home blood pressure (<135/80 mmHg) [4]. To avoid misclassifying WCHT as hypertension, blood pressure is measured at least three separate times and is often accompanied by ambulatory blood pressure measurement (ABPM) or home blood pressure measurements (HBPM), but this is still a problem, and patients may be misdiagnosed and unnecessarily treated for hypertension [9,10]. However, WCHT can be a sign of deteriorating health, and it is often accompanied by hyperlipidaemia, elevated fasting glucose levels and a tendency towards being overweight; it is also associated with a long-term risk for cardiovascular diseases and total mortality [11,12]. Evidence-based data on the therapeutic management of WCHT are lacking, but these patients should be scheduled for regular follow-up at annual visits, and recommendations for lifestyle changes should be made [4]. Therefore, it is important to identify persons with WCHT.

The overall prevalence of WCHT in the general population is between 12–15%, and among patients with increased clinical blood pressure (clinical BP), the prevalence ranges from 30–50% because of varying diagnostic criteria and BP cut-off readings [4,10,12–14]. The prevalence is lower when the office BP is based on repeated measurements or when measured by a nurse or another healthcare provider [15]. WCHT has not been previously studied in the dental setting. As dental anxiety/fear is a common condition, WCHT might be more common if BP is measured at a dental visit [16].

Several investigators have tried to identify possible determinants of WCHT, and female sex and older age have been suggested as possible predictors [4,13]. Most studies have been based on clinical BP in primary care and with a 24-h ABPM as a reference [10,13]. As dental care services are a possible provider of opportunistic BP screening and WCHT is not studied in this setting, the purpose of this study was to identify WCHT in a healthy population through an opportunistic blood pressure screening programme at dental healthcare visits along with associated risk factors.

## Materials and methods

### Design and study participants

In this multicentre study, 2025 participants aged 40–75 years were consecutively screened for high BP at their annual dental visit at four different dental clinics in a region in southern Sweden [8]. The exclusion criteria were diagnosed hypertension, diabetes, atrial fibrillation, blood dialysis, pregnancy, or the inability to handle an automatic blood pressure device. The study recruitment lasted from October 2013 to March 2015.

### Data collection and measures

Participants were screened in two stages. In the first stage, after the dental investigation, ordinary personnel (a dental nurse or a dental hygienist, educated and well trained in BP measurements) in the dental setting measured height (cm), weight (kg) and blood pressure after 5 min of rest. Blood pressure was measured in both arms a few minutes apart with a fully automatic blood pressure device (Omron M6 Comfort, Omron Healthcare Ltd., Kyoto, Japan) [17]. Those with a systolic mean BP value ≥140 mmHg and/or diastolic mean BP of ≥90 mmHg were asked to use a home BP device (Omron M6 Comfort) twice in the morning and evening for one week. Patients with an average BP at home ≥135 systolic and/or ≥85 diastolic were referred to a PHC centre for further assessment. All participants answered a health questionnaire including questions about sex, age (year), highest education (comprehensive school, elementary school, upper secondary school, university, college, other education), tobacco use (daily smoking, snuff use or previous (yes/no)), physical activity level (none, <1, 1–<3, 3–<5, ≥5 h/week), chronic diseases (hypertension, diabetes, ischaemic heart disease, dyslipidaemia, stroke, and renal failure (yes/no)), healthcare visits last year (yes/no), and whether they had measured their blood pressure in the last year (yes/no).

### Statistical analysis

For group comparisons of categorical variables, the chi-square test was utilized. The included variables were dichotomized as follows: BMI <25 kg/m^2^, ≥25; education level ≤ upper-secondary school, >upper-secondary school; and physical activity ≤3, >3 h/week. Individuals were divided into three age groups based on age: 40–49, 50–59, and 60–75 years. Multiple logistic regression analyses were performed to assess factors independently associated with WCHT both in comparison with normotension (NT) and suspected hypertension (HT). This association is expressed as odds ratio (OR) with a 95% confidence interval (CI). The independent variables were coded as follows; female = 0, male = 1, BMI <25 kg/m^2^ = 0, ≥25 = 1, no family history of hypertension = 0, family history of hypertension =1, no daily snuff use = 0, daily snuff use = 1, no daily smoking = 0, daily smoking = 1, physical activity >3 h/week = 0, ≤3 h/week = 1. All tests were double-sided, and significance was set at 0.05. The data were analysed using IBM SPSS Statistics version 26.

## Results

**Figure 1.**
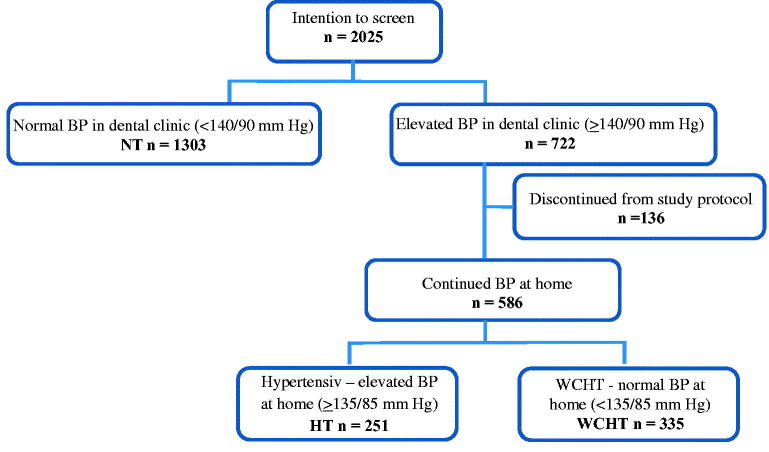
A flow chart of the study population, screened for blood pressure in dental health care in Sweden. BP: Blood pressure; NT: normotension (BP in clinic <140/90). HT: suspected hypertension (elevated average BP in dental clinic and at home). WCHT: white-coat hypertension (elevated average BP in dental clinic and normal average BP at home, both systolic and diastolic, 57.2% (335/586).

Of the 2025 people who entered the study, 1889 completed the study, and 136 (6.7%) were lost to follow-up; 1303 had a normal BP in dental clinics (<140/90 mmHg), and the remaining 722 had an elevated BP in dental clinics. Of those 586 who completed the BP measurements at home, 335 met the criteria for WCHT (57.2%), and 251 still had an elevated BP at home (Figure 1). The overall prevalence of WCHT in the study population was 17.7% (335/2025-136).

Demographic data for the study population are presented in Table 1. The mean age of the study population was 52.8 (SD 8.7), 51.6% were female, 48.4% were male, 42.4% had family history of hypertension, 38.6% had visited their healthcare provider in the past 12 months, and 43.8% had controlled their blood pressure during the last 12 months (female 43.1% and male 44.2%).

**Table 1.** Clinical characteristics, life style and education level in the study population (*n* = 2025) screened for blood pressure in dental health care in Sweden.

Variable	*n* (%)
Sex (*n* = 2025)	
Men	980 (48.4)
Women	1045 (51.6)
Age (*n* = 2025)	
40–49 years	850 (42.0)
50–59 years	704 (34.8)
60–75 years	471 (23.2)
Educational level (*n* = 2006)	
≤Upper-secondary school	1357 (67.6)
>Upper-secondary school	649 (32.4)
Body mass index, kg/m^2^ (*n* = 1976)	
<25	964 (48.8)
≥25	1012 (51.2)
Smoking (*n* = 2025)	
Daily smoking	183 (9.0)
Snuff users (*n* = 1986)	
Daily snuff users	241 (11.9)
Physical activity (*n* = 1985)	
≤ 3 h/week	831 (41.9)
> 3 h/week	1154 (58.1)

Table 2 describes the distribution of the background and lifestyle variables between the study groups. Male sex and older age were more common in the WCHT group than in the NT group (*p* < 0.0001, *p* < 0.0001) and more common in the HT group than in the WCHT group (*p* = 0.012, *p* < 0.0001). A higher educational level, BMI <25 kg/m^2^, fewer with family history of hypertension and a lower proportion of daily snuff use distinguished the NT group from the WCHT group (*p* = 0.004, *p* < 0.0001, *p* = 0.015, *p* < 0.0001). Daily smoking and low physical activity were more common among those with HT than among those with WCHT (*p* < 0.005, *p* < 0.032) (Table 2).

**Table 2.** Comparison of background and life style variables between those with normotension (NT), those with white-coat hypertension (WCHT), and those with suspected hypertension (HT).

	NT (*n* = 1303)	WCHT (*n* = 335)	HT (*n* = 251)	*p*-Value^a^	*p*-Value^b^
	*n* (%)	*n* (%)	*n* (%)	NT-WCHT	WCHT-HT
Sex				<0.0001	0.012
Men	543 (41.7)	190 (56.7)	168 (66.9)		
Women	760 (58.3)	145 (43.3)	83 (33.1)		
Age years				<0.0001	<0.0001
40–49	629 (48.3)	120 (35.8)	51 (20.3)		
50–59	434 (33.3)	121 (36.1)	103 (41.0)		
60–75	240 (18.4)	94 (28.1)	97 (38.7)		
Educational level				0.004	0.084
≤Upper-secondary school	646 (50.1)	195 (59.1)	164 (66.1)		
>Upper-secondary school	643 (49.9)	135 (40.9)	84 (33.9)		
Body mass index kg/m^2^				<0.0001	0.300
<25	754 (57.9)	126 (37.6)	84 (33.5)		
≥25	549 (42.1)	209 (62.4)	167 (66.5)		
Family history of hypertension				0.015	0.309
Yes	529 (40.6)	161 (48.1)	110 (43.8)		
No	774 (59.4)	174 (51.9)	141 (56.2)		
Healthcare visits in the past 12 months				0.129	0.850
Yes	515 (39.8)	117 (35.2)	90 (36.0)		
No	779 (60.2)	215 (64.8)	160 (64.0)		
Have blood pressure check-up in the last 12 months				0.326	0.377
Yes	572 (44.1)	137 (41.1)	112 (44.8)		
No	724 (55.9)	196 (58.9)	138 (55.2)		
Daily smoking				0.980	0.005
Yes	101 (7.9)	26 (7.8)	37 (15.2)		
No	1182 (92.1)	306 (92.2)	206 (84.8)		
Daily snuff use				<0.0001	0.161
Yes	126 (9.8)	58 (17.5)	32 (13.2)		
No	1154 (90.2)	274 (82.5)	211 (86.8)		
Physical activity				0.376	0.032
≤3 h/week	542 (42.2)	130 (39.5)	117 (48.6)		
>3 h/week	742 (57.8)	199 (60.5)	124 (51.4)		

^a^*p*-Value refers to the comparison between those with WCHT and NT.

^b^*p*-value refers to the comparison between those with WCHT and HT.

Male sex (OR 1.56, CI 1.18–2.06), older age 50–59 years (OR 1.49, CI 1.10–2.01), 60–75 years (OR 2.33, CI 1.66–3.26), family history of hypertension (OR 1.61, CI 1.24–2.10), BMI ≥25 kg/m^2^ (OR 2.36, CI 1.80–3.10), and daily snuff use (OR 1.74, CI 1.19–2.53) were all independently associated with WCHT compared to NT, as determined by logistic regression analysis (Table 3).

**Table 3.** Factors associated with white coat hypertension (WCHT), *n* = 312 compared to reference normotension (NT), *n* = 1198.

	OR	*p*-Value	95% CI
Sex	1.56	0.002	1.18–2.06
Age		<0.0001	
40–49 years	Ref.		
50–59 years	1.49	0.010	1.10–2.01
60–75 years	2.33	<0.0001	1.66–3.26
Family history of hypertension	1.61	<0.0001	1.24–2.10
Body mass index, kg/m^2^	2.36	<0.0001	1.80–3.10
Daily snuff use	1.74	0.004	1.19–2.53

Odds ratio (OR), *p*-value and 95% confidence interval (CI) by logistic regression. Controlled for daily smoking, and physical activity. The number of participants in the study (WCHT = 335, NT = 1303) is reduced in the analysis due to missing values.

Male sex (OR 2.16, CI 1.44–3.25), older age 50–59 years (OR 2.40, CI 1.52–3.81), 60–75 years (OR 2.85, CI 1.75–4.65), daily smoking (OR 2.10, CI 1.14–3.85), and less daily snuff use (OR 0.59, CI 0.34–0.99) were all independently associated with HT compared to those with WCHT, as determined by logistic regression analysis (Table 4).

**Table 4.** Factors associated with white coat hypertension (WCHT), *n* = 312 compared to reference suspected hypertension (HT), *n* = 220.

	OR	*p*-Value	95% CI
Sex	2.16	<0.0001	1.44–3.25
Age		<0.0001	
40–49 years	Ref.		
50–59 years	2.40	<0.0001	1.52–3.81
60–75 years	2.85	<0.0001	1.75–4.65
Daily smoking	2.10	0.017	1.14–3.85
Daily snuff use	0.59	0.049	0.34–0.99

Odds ratio (OR), *p*-value and 95% confidence interval (CI) by logistic regression. Controlled for body mass index, hereditary hypertension, and physical activity. The number of participants in the study (WCHT = 335, HT = 251) is reduced in the analysis due to missing values.

## Discussion

### Summary and principal findings

The main results of this study were that 17.7% of a healthy population exhibited WCHT, and 57.2% of those with an elevated BP in dental clinics had a normal BP at home. Individuals with WCHT had more cardiovascular risk factors (male sex, older age, family history of hypertension, BMI ≥25 kg/m^2^, and daily snuff use) than normotensive individuals. In comparison with WCHT, suspected hypertension was associated with male sex, older age, daily smoking, and less daily snuff use.

### Strengths and limitations

The large consecutively selected study population is a major strength of this multicentre study, which reduces the risk of selection bias. In addition, 93.3% fulfilled the study, and only 6.7% were lost to follow-up. To our knowledge, this is the largest screening-based study covering this topic. No other study has studied WCHT from an opportunistic screening perspective in ‘healthy’ patients, and among those who have studied WCHT, most have used ABPM as a reference method [10,12,18]. A limitation could be that HBPM was used as a reference, while ABPM is the ‘gold standard’ for assessing the risks of cardiovascular complications in hypertension. However, both ABPM and HBPM are recommended to confirm white-coat hypertension [4], and the strength of this study is that all study teams used a proven reliable automatic blood pressure device. Additionally, BP at home provides a greater number of readings when automatic devices are used, thereby reducing the risk of measurement bias. Another weakness is that there was only one occasion for BP measurement in dental clinics. However, on this occasion, four BPs were taken, two in each arm. Another weakness is that only one size cuff was used (for practical reasons) in the measurements performed (BP in dental clinic and BP at home), which may have affected the BP values depending on arm size. There may be a risk of false high BP for those with thick arms (BMI >30 kg/m^2^, *n* = 218) and false low BP among those with thin arms (BMI <20 kg/m^2^, *n* = 58). The BP variation for males and females is between 3–6 mmHg and 1–4 mmHg, respectively, if the wrong cuff size is used [19]. This weakness may have resulted in some false positive values and even some false negative values. Another weakness of the study is that the questionnaire was not validated in terms of background factors. The questions were simple in their formulations with dichotomous response options, which reduced the risk of misinterpretation, and therefore, we expect good face validity.

### Discussion of findings and existing literature

The prevalence rates of WCHT in an untreated population were 17.7% overall in this study and 57.2% among those with an elevated BP in dental clinics, which is slightly higher but equivalent to that in the Finn-home study, which had a similar design and study population [20]. The variable prevalence (12.1–53.2%) reported in the literature is probably explained by different study populations (such as a representative community sample or those referred to ABPM for suspected persistent hypertension), different inclusion criteria (untreated, treated, or mixed), differences in out-of-office BP monitoring (ABPM and home BP) and differences in study design [4,10,14,15,20].

Our results indicate that male sex, older age, high BMI, family history of hypertension, and daily use of snuff were associated with WCHT in comparison with NT. Male sex was also found to be more frequent among participants with WCHT in the Finn-home study [20]. Older age has been associated with WCHT vs NT in previous studies, while high BMI has not [13,20]. In the present study, male sex, older age, daily smoking and less daily use of snuff were characteristic of HT vs. WCHT. The association with male sex, older age and daily smoking has been previously shown [4,13]. A higher frequency of daily use of snuff among participants with WCHT compared to participants with NT and HT has not been previously described. The smoking frequency is low in Sweden (7% for both sexes), but many (11%), especially men (18%), use snuff instead [21]. Studies have shown that smoke-free tobacco use causes higher levels of nicotine in both serum and saliva than cigarette use [22]. As the nicotine component in tobacco causes hypertension [23], this finding is contradictory and needs to be verified in other surveys.

Patients with WCHT have a greater prevalence of metabolic risk factors, more frequent asymptomatic cardiac and vascular damage, and a greater long-term risk of progression to sustained hypertension [12]. In the progression from NT to hypertension, WCHT seems to be a middle group, which is seen in the present study regarding the associated risk factors: male sex, older age, low education, high BMI kg/m^2^, and family history of hypertension.

### Meaning of the study

Dental care providers are existing health care providers that regularly check even the healthy part of the population, and as WCHT is not more frequent in this setting than in other clinical settings, they could be potential providers for opportunistic blood pressure screening. Identifying patients with WCHT and other cardiovascular risk factors is important because they are in a state between normotension and hypertension, and it is recommended that they be checked regularly and that treatment should consider lifestyle changes, specifically targeting high BMI and tobacco use, to reduce the elevated cardiovascular risk. Individuals with WCHT can receive lifestyle advice in connection with dental check-ups, but follow-up and assessment of their cardiovascular risk should take place in primary care.

## References

[1] Lim SS, Vos T, Flaxman AD, et al. A comparative risk assessment of burden of disease and injury attributable to 67 risk factors and risk factor clusters in 21 regions, 1990-2010: a systematic analysis for the Global Burden of Disease Study 2010. Lancet. 2012;380(9859):2224–2260. 23245609 10.1016/S0140-6736(12)61766-8 PMC4156511

[2] Lindholm LH, Carlberg B. Moderately elevated blood pressure. A systematic literature review. Volumes1 & 2. Stockholm: the Swedish Council on Technology Assessment in Health Care 2004. SBU-rapport. 2004;170 = 1:1–514. and 170 = 2:1–248.

[3] Lindholt JS, Sogaard R. Population screening and intervention for vascular disease in Danish men (VIVA): a randomised controlled trial. Lancet. 2017;390(10109):2256–2265. 28859943 10.1016/S0140-6736(17)32250-X

[4] Williams B, Mancia G, Spiering W, Rosei EA, Azizi M, Burnier M, et al. 2018 ESC/ESH Guidelines for the management of arterial hypertension: the Task Force for the management of arterial hypertension of the European Society of Cardiology and the European Society of Hypertension. J Hypertens. 2018;36(10):1953–2041. 30234752 10.1097/HJH.0000000000001940

[5] Swedish Social Insurance Agency, Social insurance report [Internet]. [p. 74]. 2012. www.socialstyrelsen.se/publikationer2012/2012-2-2/Documents/Tandvardoch-tandhalsa.pdf.

[6] Engstrom S, Berne C, Gahnberg L, et al. Efficacy of screening for high blood pressure in dental health care. BMC PublicHealth. 2011;11:194. 10.1186/1471-2458-11-194 PMC3079645 21450067

[7] Greenberg BL, Glick M. Assessing systemic disease risk in a dental setting: a public health perspective. Dent Clin North Am. 2012;56(4):863–874. 23017556 10.1016/j.cden.2012.07.011

[8] Andersson H, Hedström L, Bergman S, et al. The outcome of two-step blood pressure screening in dental healthcare. Scand J Public Health. 2018;46(6):623–629. 29493430 10.1177/1403494818759840

[9] Mancia G, Bombelli M, Seravalle G, et al. Diagnosis and management of patients with white-coat and masked hypertension. Nat Rev Cardiol. 2011;8(12):686–693. 21826071 10.1038/nrcardio.2011.115

[10] Kario K, Thijs L, Staessen JA. Blood pressure measurement and treatment decisions: masked and white coat hypertension. Circ Res. 2019;124(7):990–1008. 30920932 10.1161/CIRCRESAHA.118.313219

[11] Huang Y, Huang W, Mai W, et al. White-coat hypertension is a risk factor for cardiovascular diseases and total mortality. J Hypertens. 2017;35(4):677–688. 28253216 10.1097/HJH.0000000000001226 PMC5338886

[12] Mancia G, Bombelli M, Cuspidi C, et al. Cardiovascular risk associated with white-coat hypertension: pro side of the argument. Hypertension. 2017;70(4):668–675. 28847891 10.1161/HYPERTENSIONAHA.117.08903

[13] Celis H, Fagard RH. White-coat hypertension: a clinical review. Eur J Intern Med. 2004;15(6):348–357. 15522568 10.1016/j.ejim.2004.08.001

[14] Noubiap JJ, Nansseu JR, Nkeck JR, et al. Prevalence of white coat and masked hypertension in Africa: a systematic review and meta-analysis. J Clin Hypertens. 2018;20(8):1165–1172. 10.1111/jch.13321 PMC8031123 29984891

[15] Gerin W, Marion RM, Friedman R, et al. How should we measure blood pressure in the doctor’s office? Blood Press Monit. 2001;6(5):257–262. 12055421 10.1097/00126097-200110000-00006

[16] Elfström ML, Lundgren J, Berggren U. Methodological assessment of behavioural problem dimensions in adult with dental fear. Commun Dent Oral Epidemiol. 2007;35(3):186–194. 10.1111/j.1600-0528.2006.00312.x 17518965

[17] Belghazi J, El Feghali RN, Moussalem T, et al. Validation of four automatic devices for self-measurement of blood pressure according to the International Protocol of the European Society of Hypertension. Vasc Health Risk Manag. 2007;3:389–400. 17969368 PMC2291343

[18] Sheppard JP, Fletcher B, Gill P, et al. Predictors of the home-clinic blood pressure difference: a systematic review and meta-analysis. Am J Hypertens. 2016;29(5):614–625. 26399981 10.1093/ajh/hpv157 PMC4829055

[19] Sprafka JM, Strickland D, Gómez-Marin O, et al. The effect of cuff size on blood pressure measurement in adults. Epidemiology. 1991;2(3):214–217. 2054405 10.1097/00001648-199105000-00010

[20] Hänninen MR, Niiranen TJ, Puukka PJ, et al. Target organ damage and masked hypertension in the general population: the Finn-Home study. J Hypertens. 2013;31(6):1136–1143. 23466942 10.1097/HJH.0b013e32835fa5dc

[21] Public health agency of Sweden. Livsvillkor & levnadsvanor [Internet]. Stockholm: Public health agency of Sweden; 2020. [updated 2020 May 22; cited 2020 Dec 07]. Available from: https://www.folkhalsomyndigheten.se/livsvillkor-levnadsvanor/andts/utveckling-inom-andts-anvandning-och-ohalsa/bruk/tobak-och-liknande-produkter/vuxnas-bruk-av-cigaretter-snus-och-e-cigaretter/.

[22] Prasad GL, Jones BA, Chen P, et al. A cross-sectional study of biomarkers of exposure and effect in smokers and moist snuff consumers. Clin Chem Lab Med. 2016;54(4):633–642. 26495926 10.1515/cclm-2015-0594

[23] U.S. Department of Health and Human Services CfDCaP, National Center for Chronic Disease Prevention and Health Promotion, Office on Smoking and, Health. How Tobacco Smoke Causes Disease: The Biology and Behavioral Basis for Smoking-Attributable Disease: A Report of the Surgeon General; 2010. 21452462

